# Moving towards effective therapeutic strategies for Neuronal Ceroid Lipofuscinosis

**DOI:** 10.1186/s13023-016-0414-2

**Published:** 2016-04-16

**Authors:** Ryan D. Geraets, Seung yon Koh, Michelle L. Hastings, Tammy Kielian, David A. Pearce, Jill M. Weimer

**Affiliations:** Children’s Health Research Center, Sanford Research, Sioux Falls, SD USA; Sanford School of Medicine at the University of South Dakota, Sioux Falls, SD USA; Department of Cell Biology and Anatomy, Chicago Medical School, Rosalind Franklin University of Medicine and Science, North Chicago, IL USA; Department of Pathology and Microbiology, University of Nebraska Medical Center, Omaha, NE USA

**Keywords:** Batten disease, Translational research, Palmitoyl-Protein Thioesterase 1, Tripeptidyl peptidase 1, Gene therapy, RNA modulating therapies, Antisense oligonucleotides, Enzyme replacement therapy, Stem cell therapy, Lysosomal modulators, Autophagy modulators

## Abstract

The Neuronal Ceroid Lipofuscinoses (NCLs) are a family of autosomal recessive neurodegenerative disorders that annually affect 1:100,000 live births worldwide. This family of diseases results from mutations in one of 14 different genes that share common clinical and pathological etiologies. Clinically, the diseases are subcategorized into infantile, late-infantile, juvenile and adult forms based on their age of onset. Though the disease phenotypes may vary in their age and order of presentation, all typically include progressive visual deterioration and blindness, cognitive impairment, motor deficits and seizures. Pathological hallmarks of NCLs include the accumulation of storage material or ceroid in the lysosome, progressive neuronal degeneration and massive glial activation. Advances have been made in genetic diagnosis and counseling for families. However, comprehensive treatment programs that delay or halt disease progression have been elusive. Current disease management is primarily targeted at controlling the symptoms rather than “curing” the disease. Recognizing the growing need for transparency and synergistic efforts to move the field forward, this review will provide an overview of the therapeutic approaches currently being pursued in preclinical and clinical trials to treat different forms of NCL as well as provide insight to novel therapeutic approaches in development for the NCLs.

## Background

Lysosomal storage disorders (LSDs), a group of rare disorders involving the accumulation of storage material in the lysosome, have been estimated to occur in approximately one in 7500 live births [1, 2]. Neuronal Ceroid Lipofuscinoses (NCLs), also referred to as Batten Disease, are a subset of lysosomal storage disorders that can arise from genetic mutations within one of 14 different genes [3]. Depending on the genetic mutation, these disorders can affect individuals ranging from infants to adults, though they are most commonly referred to as pediatric neurodegenerative diseases (reviewed in [4–6]). The frequency of NCLs is dependent on ancestry and geographical setting, with, for example, an estimated occurrence of up to one in 12,500 live births in Anglo-Saxon countries (reviewed in [7, 8]).

As previously mentioned NCLs can be caused by one of a number of genetic mutations and can onset at different ages. This genetic heterogeneity results in approximately 9 different forms of NCLs [4, 7]) with three most common forms being Classic Infantile Neuronal Ceroid Lipofuscinosis (INCL), Classic Late Infantile Neuronal Ceroid Lipofuscinosis (LINCL), and Juvenile Neuronal Ceroid Lipofuscinosis (JNCL; [4]). In addition to the more common forms of NCL, individuals can also be stricken with Classic Adult-Onset Neuronal Ceroid Lipofuscinosis, Finnish variant Neuronal Ceroid Lipofuscinosis, variant Late Infantile Neuronal Ceroid Lipofuscinosis (vLINCL), Turkish variant Neuronal Ceroid Lipofuscinosis, and Congenital Neuronal Ceroid Lipofuscinosis (reviewed in [4, 7]).

INCL is an autosomal recessive disorder caused by mutations in palmitoyl protein thioesterase 1 (PPT-1), a lysosomal serine lipase with a classical α/β hydrolase fold [4, 7, 9]. Like all NCLs, INCL primarily affects the CNS. Phenotypically this disorder presents with retinal degeneration, speech and motor deterioration, seizures, flat electroencephalogrphy recordings (EEG) by age three and premature death at the age 8–14 years [4, 7, 10, 11]. Pathologically, cells accumulate storage material known as granular osmophilic deposits (GRODs). Many mutations have been identified in the human gene that contributes to disease – 64 PPT1 mutations in 268 affected families (http://www.ucl.ac.uk/ncl/mutation.shtml and [11]). The top 12 mutations (3 or more families) account for 75 % of the cases.

LINCL is caused by genetic mutations in tripeptidyl peptidase 1 (TPP-1), which also codes for a soluble lysosomal enzyme whose substrates are unknown [4, 7, 12–15]. In this form of NCL as well as the juvenile form, neurons and other cells of the body accumulation autofluorescent storage material highly enriched in ATP synthase subunit c. LINCL age of onset is two-four years of life with presentation of seizures, blindness and progression motor decline and pathologically there is massive neuronal cell death. For LINCL, there is a steady state occurrence of ~ 400–500 patients in the United States; ~ 1000 patients in Europe; ~ 14,000 patients worldwide (*estimate assumes incidence of LINCL of 1:100,000 live births: average survival of 10 years, birth-rate of 14/1000 in US and Europe, 19.15/1000 worldwide). There are over 100 identified mutations in TPP1 – with an Arg208Stop and a c.509-1G > C slice mutation that are most common (http://www.ucl.ac.uk/ncl/mutation.shtml and [11]). Like PPT-1, there are also several late-onset variations of LINCL which are typically compound heterozygous for hypomorphic and null allele – with patients often surviving into the fifth decade of life.

JNCL is a fatal lysosomal storage disease caused by autosomal recessive mutations in the *CLN3* gene. JNCL typically presents in children between the ages of 5–10 years, initiating as blindness and progressing to seizures, motor loss and cognitive decline, with a decreased life expectancy into the late teens to early twenties [4, 7, 10, 11]. One very early indicator of disease is the activation of astrocytes and microglia in the brain of JNCL mice (CLN3 mutant lines) and human patients [16–18]. Currently, the physiological function of the CLN3 protein remains elusive, with what is known having been gleaned from CLN3 mutant neurons, yeast, Drosophila and mouse models of the disease.

**Fig. 1:**
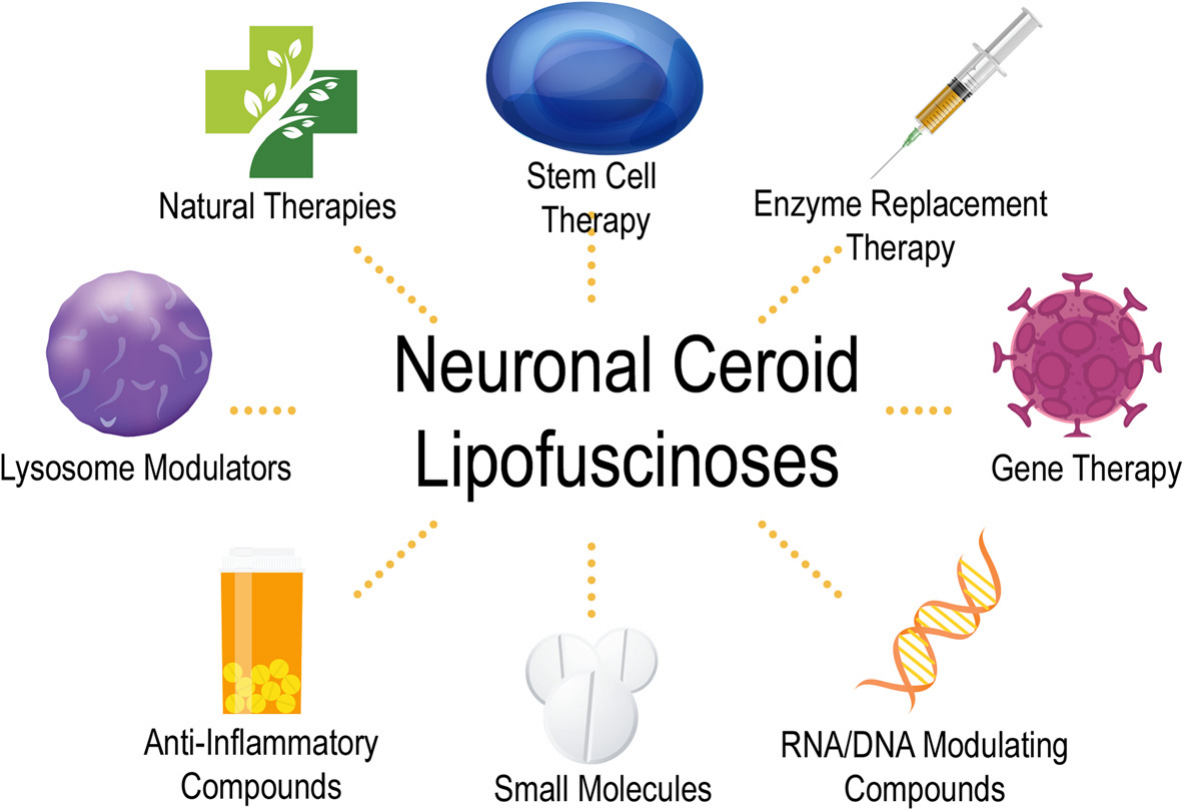
Emerging therapeutic approaches for the Neuronal Ceroid Lipofuscinoses. Diagramatic overview of therapeutic approaches being tested in preclinical and clinical trials

Nearly all forms of NCL result in death and, although a physician may explore a number of treatment strategies targeted at mitigating or controlling disease symptoms, there are currently no curative therapies. Numerous approaches are being utilized to develop potential NCL therapies. Given that each form of NCL is caused by different genetic mutations and protein deficiencies, therapeutics must be tailored specifically for each form of the disease. However, some general therapeutic strategies may be effective for different forms of NCL due to overlapping characteristics; for example, enzyme replacement therapy could be an effective approach for the forms of NCL caused by enzyme deficiencies. In this review, we summarize a number of the therapeutic approaches being used to treat different forms of this devastating disease (summarized in Fig. 1).

## Current medical management strategies for NCLs

The NCLs represent different diseases caused by mutations in as many as 14 different genes. NCLs have some common features but they are different in their clinical features, age of onset, cell biology and biochemistry, gene mutations and rate and characteristics of progression. This heterogeneity can make the discovery and use of new therapies difficult. So what treatments do we have? People often say “there are no known treatments for NCLs”. This is untrue. There are ongoing studies with anti-inflammatories that have provided some evidence of improved visual outcomes in NCLs. There are many treatments for epilepsy but very few of these have been tested specifically in NCLs. There is no known treatment for the dementia associated with NCL – although behavioral symptoms and sleep defects can mitigate symptoms to some degree. The movement disorders in NCL vary by form and, thus, so do the treatments. Myoclonus is treatable but difficult. Parkinsonism has treatment options, though ataxia is more refractory to treatment (unless those generated by vitamin deficiencies). Supportive treatment for NCL is also available– physical therapy, occupational therapy, speech therapy, feeding gastrostomy, suction and airway management and caregiver support and respite. In all, while there are treatments for NCLs, currently there are not therapies that change the outcome of the disease.

## Pipeline for drug development

Many new therapies are in the pipeline for the treatment of NCLs. Most of these may halt or slow the progression of disease but are unlikely to completely reverse the disease. Most symptomatic treatment studies in NCL have come from what we know from other diseases – but very few studies have been done specifically in NCL patients. In INCL these have been: Lamotrigine for epilepsy [19–21]; Transdermal fentanyl for pain [22]; Melatonin for sleep/circadian rhythm disturbance [23–25]; and hematopoietic stem cell transplant – umbilical cord blood and bone marrow transplant for disease modification [26]. These treatments, with a limited number of participants, are difficult to interpret because the studies are occurring at a time of rapid development, which significantly increases the variability between subjects, making outcomes difficult to measure. In LINCL, the only completed drug treatment study has been the melatonin study mentioned above. There have been a number of LINCL disease modification studies, such as bone marrow transplantation [27–29] and antioxidants (selenium, VitE, [30]). It should be noted that these antioxidant studies were completed prior to the availability of NCL molecular diagnostics. Additionally, there are a number of ongoing studies in various stages of clinical trial (Table 1) including gene therapy trials using adeno-associated viral vector (ClinicalTrials.gov, NCT00151216) and stem cell trials using human CNS derived stem cell treatment (ClinicalTrials.gov, NCT00337636), both which we will talk about more in this review. What do we need to do to accelerate this process? Better understanding of the clinical features of the different NCLs and within type variants will provide the basis for better symptomatic treatments. We need better understanding of the pathobiology of the NCLs in order to identify the best course for disease-modifying treatments. Additionally, we need to develop new delivery mechanisms – for delivery of enzymes or restoration of disease causing mutations.

**Table 1:** Past and present NCL clinical trials. Currently, there are only eight NCL clinical trials in existence (clinicaltrials.gov). Most of the trials focus on the treatment of either INCL or LINCL

Trial ID	NCL Form(s)	Therapeutic Approach	Proposed Mechanism of Action	Preclinical Studies	Trial Phase
NCT01399047	JNCL	**Anti-inflammatory** Mycophenolate mofetil	Reduction in neuroinflammation and production of autoantibodies	Seehafer 2011 [144]	Recruiting
NCT01161576	LINCL	**Gene Therapy** AAVrh.10CUhCLN2	Genetically engineer cells to produce non-mutated TPP1	Sondhi 2007 [41], Sondhi 2008, Sondhi 2012	Recruiting
NCT01414985	LINCL	**Gene Therapy** AAVrh.10CUhCLN2	Genetically engineer cells to produce non-mutated TPP1	Sondhi 2007 [41], Sondhi 2008 [42], Sondhi 2012 [43]	Recruiting
NCT01907087	LINCL	**ERT** BMN-190	Source of recombinant functional TPP1 in which diseased cell can uptake and utilize	Vuillemenot 2014 [58], Vuillemenot 2014 [59]	Active
NCT00151216	LINCL	**Gene Therapy** AAV2CUhCLN2	Genetically engineer cells to produce non-mutated TPP1	Sondhi 2005 [39], Passini 2006 [40]	Active
NCT00337636	INCL, LINCL	**Stem Cell** Human CNS Stem cells	Similar to ERT but, human CNS stem cells act as the source of functional PPT1 and TPP1	Tamaki 2009 [85]	Complete
NCT00028262	INCL	**Small Molecule** Cystagon	Clears lysosome of storage material	Zhang 2001 [174]	Complete
NCT01238315	INCL, LINCL	**Stem Cell** Human CNS Stem cells	Similar to ERT but, human CNS stem cells act the source of functional PPT1 and TPP1	Tamaki 2009 [85], Selden 2013 [91]	Withdrawn

Bold words indicate the therapeutic approach

## Gene therapy

Over the past couple of decades gene therapy has developed into a promising therapeutic treatment option for LSDs. Recently, the European Union approved its first gene therapy for the treatment of lipoprotein lipase deficiency [31]. Unlike lipoprotein lipase deficiency, NCLs are predominately neurodegenerative diseases, and thus harder to treat. When one thinks about the use of gene therapy in the treatment of any disease, including NCLs, there are a number of technical hurdles that must be overcome. As mentioned above, delivery to the primary diseased tissues is critical. In the case of NCLs, this means delivery of the virus into the central nervous system. There are a number of different viral vectors that are currently utilized for gene therapy, including as adeno-associated virus (AAV) and lentivirus (LV). A particular vector is selected based on several factors including: payload (of the size of the gene to be delivered), the region they need to be delivered to and types of cells to be targeted to, and how much of the gene needs to be expressed [32–35]. In addition, these vectors and their associated serotypes can be modified to gain additional desired properties, including the timing of expression and tropism to specific cell populations [32–35]. Thus a number of ongoing studies are focused on tailoring gene therapy vectors to various forms of LSDs and NCLs.

In terms of NCLs, a number of studies have focused on using AAV to treat both INCL and LINCL [36–41, 44–46]. Gene therapy studies done by Griffey et al. utilized AAV serotype-2 (AAV2) to treat *Ppt1*^*−/−*^ mice; collectively these studies illustrated that AAV-2 increased PPT-1 activity, reduced levels of autofluorescence, improved retinopathy and eliminated some behavioral phenotypes [36–38]. LINCL gene therapy studies have treated *Tpp1*^*−/−*^ mice with various AAV serotypes which resulted in increased Tpp1 expression, improved behavioral phenotypes and a reduction in autofluorescence [39–41]. In addition to animal studies, LINCL gene therapy clinical trials (NCT00151216 and NCT01161576) are currently ongoing (ClinicalTrials.gov) and a preliminary report from study NCT00151216 indicates that the AAV2 mediated gene therapy shows promise in reducing the rate of LINCL progression [45].

NCL gene therapy studies are not restricted to the aforementioned approaches. Recent studies have grouped gene therapy with other therapeutic approaches; for example small molecule therapies and hematopoietic stem cell therapy [46]. Also, other AAV vectors exist and have been noted to be more effective; for example self-complementary vectors and AAV9 [47–51]. Previous work with AAV9 [49, 50] and its potential use in the treatment of NCLs was discussed at the 2014 Update of Translational Research for Management of INCL/LINCL Conference. Based on the results from the NCL gene therapy studies and the conference, gene therapy has great potential for being an NCL therapeutic.

## Enzyme replacement

In addition to gene therapy, enzyme replacement therapy (ERT) is also being heavily pursued as a therapeutic approach for the treatment of LSDs. A basic search for ERT and LSDs (using the Department of Health and Humans Services (HHS) website, ClinicalTrials.gov) results in more than a hundred registered clinical trials. In a recent technical brief, it was noted that nine ERTs are available for the treatment of a limited number of LSDs within the United States; but unfortunately none are for the treatment of NCLs [52]. Nevertheless, numerous preclinical studies have been conducted using ERT to treat different forms of NCL. ERT seem promising specifically in INCL and LINCL as these forms of NCL are caused by enzyme deficiencies. But a number of studies have reported perturbation in lysosomal enzymes, including PPT-1 and TPP-1, in other non-enzyme mediated forms of the disease, suggested ERT may have more global applications. Pre-clinical ERT studies intended to treat an INCL mouse model have been limited to only two studies and have revealed the following: (1) they are able to effective produce recombinant PPT-1, (2) ERT is able to clear autofluorescent storage material in certain peripheral tissues, (3) they are able to achieve partial delivery of ERT to brain via intravenous injection, (4) treatment is able to elicit mild changes in phenotype, and (5) administration of the recombinant PPT-1 is tolerable in mice [53, 54].

In comparison to INCL, pre-clinical ERT studies for LINCL have been more extensive. These studies have used a variety of animal models of disease including mice, dogs and monkeys and have been administered using alternative delivery methods: intravenous, intrathecal and intraventricular [55–60]. Collectively, these studies have demonstrated: (1) distribution of recombinant TPP-1 includes the brain and peripheral tissues however, distribution is dependent on the method of delivery, (2) ERT provides improved disease phenotype and pathology, (3) there is a reduction in autofluorescent storage material accumulation within the brain, and (4) depending on the delivery method and other various factors, there are minimal adverse reactions associated with ERT in these animal models [55–60]. In addition, some of these studies have led to the development of an ERT clinical trial for the treatment of LINCL (NCT01907087, Clinicaltrials.gov)

Overall, ERT seems to be a promising therapeutic approach for the treatment of some forms of NCLs. As with many therapies, the blood-brain barrier (BBB) seems to be a persistent obstacle for LSDs that effect the CNS [55–68]. Given that the CNS is predominantly affected in the NCLs, methods to surpass the BBB are being addressed in current preclinical studies and were discussed at the 2014 Update of Translational Research for Management of INCL/LINCL Conference. These approaches range from direct ERT delivery to the brain via intrathecal or intraventricular injections to modifying the recombinant protein [55–60, 68]. In particular, Meng, et al., demonstrated that intravenous injections of recombinant TPP-1 could penetrate the blood-brain barrier if fused to a small recombinant section of zapolipoprotein E [60]. Given that some of these approaches are new to the field of ERT, further refinement is ongoing, even as the first ERT trials have begun in NCL patients.

## Small molecule carriers: Trojan horses, modified receptor, liposomes and nanoparticles

As previously discussed, one of the more challenging obstacles to overcome in LSD therapy development is penetrance of the blood-brain barrier. A number of approaches are currently being explored to surpass the BBB including peptide modification. In two separate studies, TPP-1 was modified by either altering the protein glycosylation profile or combining the TPP-1 peptide sequence with a specific region of the apolipoprotein E receptor and resulted in increased BBB penetrance [56, 60]. This method, sometimes referred to as a “Trojan Horse”, utilizes natural cellular pathways to deliver proteins across the BBB and into target cells [65, 69]. This method has been effectively applied to other LSDs including subtypes of Mucopolysaccharidosis [65, 67, 70, 71].

An additional strategy for effect therapeutic deliver across the BBB are nanocarriers, including liposomes (reviewed in [65, 71]). The use of this technology has also been studies in animal models of both Mucopolysaccharidosis and Niemann Pick disease, showing great potential [64–66, 71–73]. One advantage of nanocarriers is targeted cell deliver, often achieved by coating the nanoparticle with different components (including antibodies to surface proteins) in order to direct their delivery [65, 71]. This method has been successfully applied in animal models LSDs to target cell surface receptors (i.e., using antibodies to PECAM-1, transferrin receptor (TfR) and intercellular adhesion molecule 1 (ICAM-1) expressed on endothelial cells of the BBB [64–66, 71–73]. Ansari et al., has also generated similar liposomal carriers to transport cargo in NCL cell models [74], further supporting the use of both peptide modification and nanocarriers as favorable approaches to deliver NCL therapies into the CNS and to effected cells.

## Stem cell therapy

As the field of regenerative cellular therapies expands, various different types of stem cells are providing increased utility in the treatment of neurological disorders. But like with other treatment options, there are a number of technical considerations that must be taken in selecting which type of stem cells to test for use in treatment. What is the best type of cell to utilize? What is the potential of the stem cell being used to 1) enhance the immune system and/or 2) replace lost cells? How will the stem cells be delivered to the damaged tissue? These are all thing that must be considered. Some LSDs, including Hurler Syndrome, have shown potential for being treated with adipose and hematopoietic stem cells (HSC; [75–79]). A number of NCL mouse model and patient studies have explored the benefits of HSC therapy but met with limited success partially owing to the limited patient sample size [26, 28, 29]. The most promising of these studies has suggested that HSC, specifically bone marrow treatment, offered in combination with gene therapy in *Ppt1*^*−/−*^ mice significantly improved outcomes even when HSC therapy alone provided limited or no benefit. Thus, HSC should not be entirely eliminated as a potential NCL therapeutic.

Neural stem cell therapies, derived from a variety of sources, are also being studied as a therapeutic for LSDs including NCLs [80, 81]. Many of these studies utilize either murine or human neural stem cells to treat mouse models of LSDs and data from clinical trials is thus far limited. Collectively, these studies have shown: (1) neural stem cells can survive within the CNS, (2) they are capable of migrating away from site of injection, (3) they can improve disease pathology, including dampening neuroinflammation, (4) increase enzyme activity, (5) and improve long-term survival [80–90]. However, the effectiveness seems highly variable depending on the type of LSD and other compounding factors. Tamaki et al., showed improved disease outcomes in a *Ppt1*^*−/−*^/NSCID mouse model following treatment with human CNS derived stem cells and these findings resulted in a clinical trial (ClinicalTrials.gov, NCT00337636; [85]). The outcomes from this trial point toward successful transplantation of CNS derived stem cells into both INCL and LINCL patients ([91]) and thus merit continued exploration as a possible treatment.

One source of stem cells yet to be explored as a treatment option for NCLs are induced pluripotent stem cells (iPSCs). Since being first described in 2006 by Takahashi and Yamanaka, iPSCs have been used for various research purposes [92, 93]. A limited yet expanding number of studies have focused on using iPSCs for therapeutic purposes [93–96]. Given their potential as a therapy, the use of iPSCs as a therapeutic approach for NCLs is now being considered by a number of research laboratories.

## RNA modulation

RNA modulation therapies are a relatively new therapeutic approach for lysosomal storage diseases, especially the NCLs. There are a number of different RNA modulation therapies [i.e., antisense oligonucleotides (ASO), nonsense suppression compounds, nonsense mediated decay (NMD) inhibitors] that have been used effectively in preclinical and clinical trials for a number of different diseases [97–111]. These therapies use different strategies to reach a similar end-goal – that of producing a partially or fully functional protein from the targeted mRNA transcript.

One therapeutic strategy aimed at treating diseases caused by nonsense mutations involves the use of nonsense suppression compounds which promote the readthrough of nonsense or premature termination codons (PTCs). A proportion of PTC-containing transcripts escape NMD and these compounds induce the ribosome to incorporate a near cognate amino acid at the PTC, essentially “reading through” the termination codon. For these mRNAs, translation continues through the entire transcript, terminates at the natural termination codon and thus generates a full-length protein with a single amino acid substitution at the nonsense mutation. Read-through therapy, or nonsense suppression therapy, is currently in clinical trials (Clinicaltrials.gov, NCT00264888, NCT00592553, NCT01826487, NCT00237380, NCT01140451, NCT01918384), [112, 113]).

Can these therapies be effectively applied to the NCLs? Nonsense suppression therapies have been proposed as treatments for a whole host of LSDs that result from nonsense mutations. In fact, nonsense mutations in *CLN1* are present in more than 50 % of INCL patients [114]. Multiple studies have used different read-through compounds to treat several models of LSDs, including INCL and LINCL [97–99, 102, 104, 107, 108]. Specific to the NCLs, gentamicin, an aminoglycoside, can been used as a nonsense suppression therapy and can increase TPP-1 activity in LINCL fibroblasts [98]. Moreover, two recent studies have demonstrated the potential effectiveness of Ataluren (PTC124; currently in clinical trials for Duchenne Muscular Dystrophy (Clinicaltrials.gov; NCT00264888, NCT00592553, NCT01826487) and cystic fibrosis (Clinicalrials.gov; NCT00237380, NCT01140451)) as a nonsense suppression therapy for INCL and LINCL with both reports indicating increases in enzyme activity after treatment of patient-derived lymphoblast cell lines [102, 107]. The potential utility of PTC-124 was further illustrated in vivo using a recently developed *Cln1*^R151X^ mouse model [108] which also showed elevated enzyme activity levels over untreated mutant mice. Although PTC-124 has the advantage of being orally bioavailable and has low toxicity, it has a narrow therapeutic window and short half-life [115] – so patients may need to be treated as much as three times per day, which limits its practical use in patients. However, a number of additional nonsense suppression therapies are currently being developed which aim to improve therapeutic efficacy. Additionally, a number of teams are starting to combine nonsense suppression therapies with other treatments. For example, Keeling et al. has shown that combining read-through compounds with nonsense mediated decay (NMD) inhibitors intensifies nonsense suppression [106], broadening the possibilities for another potential avenue of NCL therapies.

Another RNA modulating therapeutic approach is the use of antisense oligonucleotides (ASOs). These short, modified nucleic acids are designed to bind a target RNA through complementary base-pairing [103]. The binding of an ASO to RNA can modify RNA processing by sterically blocking the binding of RNA binding proteins. ASOs have many favorable drug-like features including low toxicity, easy deliverability to a wide-range of cells in vivo, and stability, with activity in cells lasting up to a year after a single dose [110]. Several ASO drugs are used in the clinic and many others are in clinical trials for a number of diseases and conditions including the pediatric neurodegenerative disease spinal muscular atrophy and Duchenne’s muscular dystrophy [103, 110, 116] as well as a number that are being utilized in preclinical studies, include one for the pediatric neurosensory disorder Usher syndrome [117] and in the lysosomal storage disorder, Niemann-Pick Type C [101]. Depending on the genetic mutation, ASOs could potentially be used to treat various forms of NCLs. Overall, RNA modulation therapies are a new and expanding therapeutic approach and have great potential as a therapy for NCL.

**Fig. 2:**
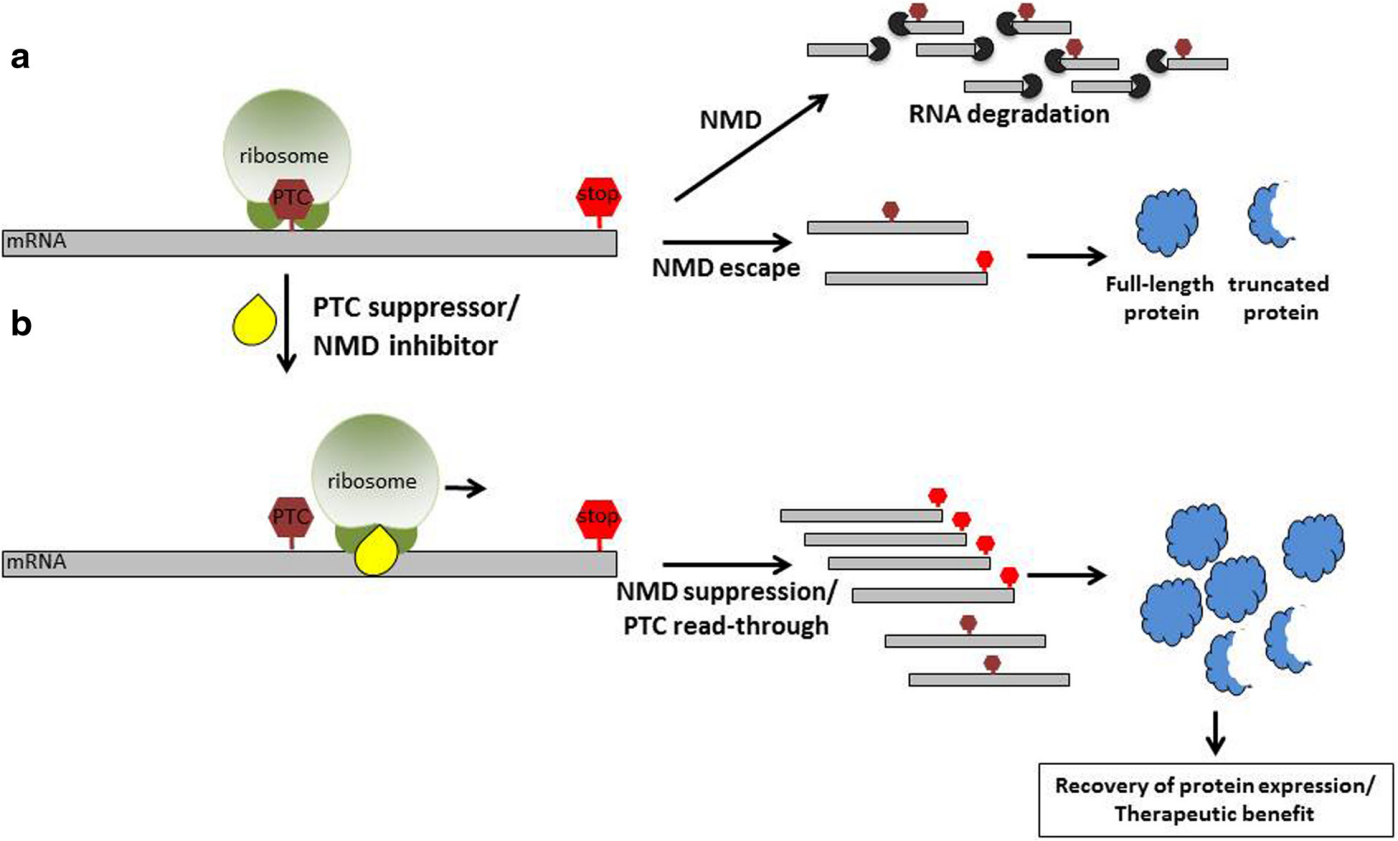
Nonsense suppression therapies in combination with nonsense mediated decay inhibitors. **a** Transcripts containing premature termination codons (PTC) are targeted for degradation via nonsense mediated decay (NMD) resulting in decreased protein production. However, transcripts that escape NMD predominately lead to the translation of truncated proteins. **b** The negative effect of PTCs, truncated protein production, can be suppressed with PTC suppressors and NMD inhibitors. NMD inhibitors prevent NMD resulting in a larger portion of PTC containing transcripts. PTC suppressors promote the translation of PTC containing transcripts into full-length proteins. When PTC suppressors and NMD inhibitors are utilized in combination, the outcome is synergistic thus resulting in an increased abundance of full-length proteins

ASOs may be useful in combination with nonsense suppression therapies to improve the protein production from genes with nonsense mutations. A limitation to the nonsense suppression approach is the activity of the naturally occurring process in eukaryotic cells called nonsense mediated decay (NMD; Fig. 2) [118]. NMD maintains RNA fidelity by eliminating mRNA transcripts that have PTCs [119]. In this way, NMD prevents the production of aberrant, truncated proteins. However, at the same time, by eliminating mRNA with PTCs, NMD also limits the amount of mRNA that can be targeted by nonsense suppression drugs for translational read-through and full-length protein production Miller Pearce. In order to overcome this limitation, small molecule inhibitors of NMD have been explored as potential therapeutic compounds [120]. The rationale for using NMD inhibitors as a treatment for diseases caused by nonsense mutations is that by making NMD less efficient, the abundance of mRNA that is translated will increase, which will increase the efficacy of nonsense suppression drugs. Recently, Krainer and colleagues demonstrated that ASOs that basepair at specific sequences of a PTC-containing pre-mRNA, were able to protect the mRNA from NMD [121]. When used in combination with readthrough compounds, the ASO increased full-length protein production from the nonsense-mutant allele more than the readthrough compound alone. A similar approach can be envisioned to aid in the efficacy of nonsense suppression approaches for NCL.

## Anti-inflammatories

Perturbation in normal inflammation response has long been suspected as an integral part of the pathobiology of a number of neurodegenerative diseases including lysosomal storage disorders [122–129]. It appears that neuroinflammation in LSDs can encompass numerous components such as, alterations in inflammatory associated gene expression, adjustments in cytokine levels, microglia activation, lymphocyte infiltration and production of auto-antibodies; however, not all LSDs display the same components [16, 17, 123, 125–147]. Some if not all of the aforementioned constituents of neuroinflammation have been shown to play a role in the pathogenesis of NCLs: INCL [130, 131, 138, 140, 142, 143, 145, 148–150]; LINCL [150–152] JNCL [16, 17, 133, 134, 139, 144, 146, 147, 150]; CLN5 [150, 153, 154]; and CLN6 [136, 137].

Based on the fact that inflammation is involved in NCL disease progression, the use of anti-inflammatories as a therapeutic approach has been addressed. Mycophenolate mofetil, an immunosuppressant, when used in *Cln3*^*−/−*^ mice appeared to protect against neuroinflammation, deposition of immunoglobulin G in the brain, and neuronal cell death [144] and these findings contributed to an ongoing JNCL clinical trial (NCT01399047; Clinicaltrials.gov). In contrast, the use of another anti-inflammatory, minocycline, in a *Cln6* ovine model did not alter disease pathology [136]. Additionally, a number of scientists are actively pursuing the use of anti-inflammatories to target components of JNCL associated neuroinflammation. Collectively, these studies indicate that the success of anti-inflammatories may be dependent on the form of NCL, and since anti-inflammatories do not address the underlying cause of NCLs, these therapies may function best if used in combination with other treatments.

## Lysosomal modulators

Lysosomal storage disorders result from a deficiency in either a soluble lysosomal enzyme or a lysosomal transmembrane protein, which can result in the accumulation of lysosomal storage material. One obvious approach is in modulating the lysosome to promote the clearance of lysosomal storage material. Sardiello et al. have previously reported that TFEB, a transcription factor, can modulate the lysosome by altering the expression of a number of lysosomal genes [155]. Since then, Palmieri et al. have clarified the genetic targets of TFEB which include various *CLN* genes [156]. Due to the effect of TFEB activation on lysosomal genes, TFEB has become a therapeutic target for lysosomal storage disorders in general, including the NCLs [157–162]. Studies have identified a number of TFEB activators; one of which reduces storage accumulation in LINCL patient fibroblasts [155, 161, 162]. Considering TFEBs targeting of *CLN* genes and the recently identified activators of TFEB, lysosomal modulation via TFEB appears to be a viable therapeutic approach for NCLs. In addition to TFEB activators, other compounds have been shown to modulate the lysosome of LSDs, including, δ-tocopherol [163]. It is important to note that therapeutics based on lysosomal modulation do not address the underlying cause of NCLs and thus may be more suitable as a combinatorial therapy.

## Small molecules and alternatively targeted pathways

In addition to the molecules previously discussed, a number of newer small molecule compounds are just beginning to be tested for their therapeutic potential in NCLs. Recently, CRMP2 has been associated with neurodegenerative diseases including the NCLs [164–166]). Due to this association, the targeting of CRMP2 utilizing various compounds (i.e. LKE, lacosamide) may be a therapeutic option for NCLs [165–172]. In addition, NtBuHA, a hydroxylamine derivative, was screened by Sarkar et al. in INCL cell lines and a mouse model; results indicate improved disease associated phenotypes, such as storage material and neurodegeneration [173]. Lastly, the small molecule compounds cysteamine bitartate and N-acetylcysteine have been assessed using models of INCL, which lead to the clinical trial (Cinicltrials.gov, NCT00028262 [174–176])

Based on the aforementioned compounds and their results, small molecules seem to be a potential therapeutic option for the different forms of NCL. In order to identify new small molecule therapies, high throughput drug screens (HTS) have been used with some success in a number of LSD studies to screen various compound libraries [177–179]. Overall, both novel and established small molecule compounds could be uncovered through these studies. However, depending on the compounds mechanism of action they too may function best as a combinatorial therapy.

## Natural treatments (ex. Antioxidents, selenium, VitE, curcumin)

Analysis of natural compound treatments (i.e., Vitamin E, selenium) for NCLs began in the late 20th century. Naidu et al., presented these studies in their report discussing the use of selenium in three cases of NCL [30]. Since then, various studies have focused on using this therapeutic approach in NCL models. The following are just a few examples: antioxidants Vitamin E [180] and Resveratrol [181–183]; endoplasmic reticulum modifiers TMAO [184] and TUDCA [184]; and NtBuHA [173]. Particularly, resveratrol has shown beneficial effects when used to treat both INCL and JNCL cell lines in addition to *Cln1* knockout mice [181–183]. Even though different homeopathic treatments have demonstrated promising results, they too only address secondary consequences and not the underlying cause of NCLs. Therefore, in addition to lysosomal modulators, this therapeutic approach may function well as a combinatorial therapy.

## Conclusions

As the various treatment options that we have reviewed are being explored, we must simultaneously ensure that we have a strong foundation on which to successfully accelerate these treatments into and through clinical trials. This includes making sure that we have the necessary tools in place to expedite these studies including comprehensive natural history studies of the NCLs, detailed and easy-to-use clinical rating scales, systems for early diagnosis (including clinical education and distance medicine for virtual diagnosis), and reliable biomarkers for tracking disease progression. Our clinical and basic research teams must be well trained in the ethical and management issues involved in conducting clinical trials. Additionally, even at the earliest stages of pre-clinical work in animal models of the NCLS, the rigor of clinical research must be applied. Failure to maintain a high level of rigor can result in our wrongly advancing (or dropping) therapeutic targets, unnecessary higher costs of production, unknown risks/benefits, ethical concerns pertaining to risking patients with invalid candidate therapeutics, and/or late state trial failures that in turn prevent patients from being involved in other trials. Thus, a number of reports from the NIH and the pharmaceutical industry have stressed the growing importance of rigor in how preclinical studies are designed and executed in order to optimize the predictive value of preclinical studies [185]. How do we, as members of the research community ensure that this is happening so that we can collectively accelerate treatments for the NCLs? We need to design our studies with increased rigor, including blinding our research staff and randomization of subject group assignment, and ensure that these details are transparent in grant proposal and research reports. Based on the numerous therapeutic approaches discussed here and their potential as treatments of NCLs, these factors have to be strongly considered due to the limited number of patients and the ultimate goal of identifying curative therapeutics.

We must also all acknowledge that although this review focuses on a number of potential NCL therapies, it does not include every possibility. In addition to drawing on the current literature to identify areas of current and potential focus for therapy development, the authors of this review are also very active in the NCL research community and attend a number of research forums focused on these topics. In particular, a portion of the topics discussed here and opinions on these potential therapeutic approaches are highlights from a periodic forum held between member of the research community, patient advocates and public policy group entitled Batten Disease: Updates on Translational Research for Management of INCL/LINCL. However, the field of translational research is moving at an accelerated pace and we as research scientist, clinical and regulatory officials must work collectively to streamline efforts that effectively and efficiently allow new drugs and treatment strategies to move from the basic laboratory to the patient as quickly as possible.

## Abbreviations

ASO: antisense oligonucleotide
BBB: blood brain barrier
CNS: central nervous system
ERT: enzyme replacement therapy
GRODs: granular osmophilic deposits
HSC: hematopoietic stem cell
INCL: Infantile Neuronal Ceroid Lipofuscinoses (INCL)
iPSC: induced pluripotent stem cell
JNCL: Juvenile Neuronal Ceroid Lipofuscinoses
LINCL: Late Infantile Neuronal Ceroid Lipofuscinoses
NCL: Neuronal Ceroid Lipofuscinosis
NMD: nonsense mediated decay
PPT1: palmitoyl-protein thioesterase 1
TPP1: tripeptidyl peptidase 1
